# Is Brazil Reversing the Decline in Childhood Immunization Coverage in the Post-COVID-19 Era? An Interrupted Time Series Analysis

**DOI:** 10.3390/vaccines13050527

**Published:** 2025-05-15

**Authors:** Ramon Costa Saavedra, Rita Carvalho-Sauer, Enny S. Paixao, Maria Yury Travassos Ichihara, Maria da Conceição Nascimento Costa, Maria da Glória Teixeira

**Affiliations:** 1 Bahia State Health Department, Salvador 41745-900, BA, Brazil; ritacarvalhosauer@gmail.com; 2 London School of Hygiene and Tropical Medicine, London WC1E 7HT, UK; enny.cruz@lshtm.ac.uk; 3 Center for Data Integration and Knowledge for Health—CIDACS, Salvador 41745-715, BA, Brazil; maria.yury@fiocruz.br; 4 Institute of Collective Health, Federal University of Bahia, Salvador 40110-040, BA, Brazil

**Keywords:** COVID-19 pandemic, vaccination coverage, childhood vaccination, interrupted time series

## Abstract

**Background**: The COVID-19 pandemic had significant impacts on healthcare systems, including the disruption of essential services such as childhood immunization. Containment measures, such as social distancing, contributed to reduced adherence to vaccination programs, increasing the risk of re-emerging vaccine-preventable diseases. We aim to assess the evolution of childhood vaccination coverage in Brazil from 2010 to 2024, identifying trends before, during, and after the COVID-19 pandemic. **Methods**: An interrupted time series (ITS) study was conducted using publicly available aggregated data on vaccination coverage for children under one year of age. Prais–Winsten regression models were applied to estimate trend changes and evaluate the impact of the pandemic on immunization levels. **Results**: The findings indicate a progressive decline in vaccination coverage between 2010 and 2019, which was intensified in 2020 by the pandemic. The BCG vaccine showed the greatest decline (−24.88%, *p* < 0.001), while pentavalent and hepatitis B vaccines decreased annually by −3.72% and −2.21%, respectively. From 2021 onwards, a gradual recovery in coverage was observed, with significant increases for BCG (+7.48% per year, *p* < 0.001), hepatitis B (+7.45%, *p* = 0.014), and MMR (+6.73%, *p* = 0.017) vaccines. **Discussion**: The results highlight a concerning decline in childhood immunization, exacerbated by the pandemic but showing recent signs of recovery. This scenario underscores structural challenges within the National Immunization Program, requiring coordinated efforts to reverse vaccination losses and ensure system resilience in the face of future crises.

## 1. Background

The COVID-19 pandemic posed one of the greatest global health challenges of the 21st century, exposing significant weaknesses in healthcare systems. Beyond its direct consequences, the health crisis triggered profound collateral effects, including the disruption of essential services such as immunization programs. Measures such as social distancing and the suspension of non-emergency activities, while crucial for containing the spread of SARS-CoV-2, led to an alarming reduction in the use of routine healthcare services, with particularly concerning consequences for vaccination activities [1,2].

This critical scenario may have negatively impacted vaccination coverage rates, exposing communities to the risk of re-emerging vaccine-preventable diseases. In the context of child health, this situation is particularly worrisome given the essential role vaccines play in reducing morbidity and mortality during this stage of life [3,4].

Vaccines are among the most effective and cost-efficient public health interventions, saving millions of lives and significantly contributing to increased life expectancy on a global scale. Compared to a scenario without vaccination, immunization prevents exorbitant expenses related to hospitalizations, treatments, and diagnostic tests, establishing itself as an essential pillar for the sustainability of healthcare systems [5,6].

In Brazil, a country recognized for its highly effective National Immunization Program (NIP), established in 1973, historical advances have been achieved through mass vaccination campaigns and the implementation of a robust immunization schedule. These efforts have led to the control, elimination, and even eradication of diseases such as measles, polio, and smallpox [6,7]. However, in the second decade of the 21st century, this progress stagnated, followed by a persistent decline in vaccination coverage, particularly for childhood immunizations. In 2019, for the first time in history, the country failed to meet coverage targets for any of the vaccines recommended for children under one year of age [8,9].

This decline can be attributed to multiple factors, including a false sense of security among the population. As previously controlled diseases became less visible, public perception of the importance of vaccination diminished [10,11]. Additionally, misinformation, amplified by anti-vaccine movements and the spread of fake news on social media, has played a central role in vaccine hesitancy, a phenomenon recognized by the World Health Organization (WHO) as one of the top ten global health threats [12,13,14].

In this context, the declaration of a public health emergency due to the novel coronavirus in Brazil in March 2020 posed an additional challenge to an already fragile immunization landscape [15,16]. Drastic changes occurred within the healthcare system, including service disruptions and decreased demand for preventive care such as vaccination. This paradoxical scenario—characterized by low adherence to routine vaccines alongside heightened anticipation for COVID-19 vaccines—raises concerns about the potential resurgence of previously controlled diseases such as measles, which had already seen localized outbreaks even before the pandemic [8,17].

Given this reality, it is fundamental to systematically evaluate the impact of the COVID-19 pandemic on childhood vaccination coverage, identify the factors associated with declining immunization rates, and propose strategies to mitigate these effects. By addressing this globally relevant topic, the present research aims to provide evidence for developing guidelines that bolster the resilience of immunization programs in the face of future health crises, thereby protecting vulnerable populations and ensuring the sustainability of healthcare systems.

Specifically, this study analyzes trends in childhood immunization coverage in Brazil over the decade 2010–2019 and examines the magnitude and temporal evolution of these rates since the onset of the COVID-19 pandemic (2020–2024). In addition, we discuss factors that may have contributed to these trends, with the goal of informing public policies aimed at preventing vaccine-preventable diseases and enhance the resilience of the National Immunization Program in the face of future crises. In addition, we discuss factors that may have contributed to these trends, with the goal of informing public policies aimed at preventing vaccine preventable diseases and enhance the resilience of the National Immunization Program in the face of future crises.

## 2. Methods

### 2.1. Study Design

An interrupted time series (ITS) study was conducted using publicly available aggregated data on routine vaccination coverage recommended for children up to one year of age. ITS allows for the longitudinal analysis of data before and after a disruptive event to identify changes in the levels and trends of outcome variables at the population level [18].

### 2.2. Data Sources and Study Period

Vaccination coverage data were obtained from the National Immunization Program Information System, where records of administered vaccine doses in Brazil are entered [19]. The NIP, one of the largest vaccination programs in the world, is funded with resources from government tax collection. Therefore, all vaccines analyzed in this study are provided free of charge to anyone seeking healthcare in Brazil [6,7,20].

Parents know which vaccines they need to give their babies up to 1 year old through the National Child Vaccination Calendar, which is widely disseminated by the Brazilian Ministry of Health. It indicates the types of vaccine, vaccination schedules, and the period in which each vaccine should be administered. In addition, immunization actions are part of the routine activities of Primary Health Care in Brazil, where health teams monitor the evolution of the population’s vaccination records and advise parents on the need for updates. For the BCG and hepatitis B vaccines, the recommendation is that they be administered in maternity wards, in the first days of the child’s life. However, if this is not the case, they can be administered in one of the more than 40,000 basic health units, together with the other vaccines recommended for children under 1 year old analyzed in this study [20].

If a child misses the recommended period to receive a certain vaccine, there is a grace period for the application of delayed vaccine doses. This period varies according to each type of vaccine, as follows: hepatitis B (can be administered up to 30 days of age); human rotavirus (first dose up to 3 months and 15 days of age; second dose up to 7 months); BCG, polio, pneumococcal 10v, meningococcal C, yellow fever and triple viral (can be administered up to before the child turns 5 years old), pentavalent (can be administered up to before the child turns 7 years old) [20].

In our study, the analysis period covered the years from 2010 to 2024, except for the meningococcal C conjugate and pneumococcal vaccines, which were analyzed from 2011, and the pentavalent vaccine, from 2013, due to the lack of previous data. For the year 2024, data refer to the period up to October, the last available month at the time of data extraction for this study in January 2025. The pre-pandemic period (2010–2019) served as the baseline, while the pandemic and post-pandemic period (2020–2024) was used to assess potential changes in vaccination coverage.

### 2.3. Study Population and Area

The study population comprised live births in Brazil between 2010 and 2024, averaging 2.7 million per year. Brazil has a territorial area of 8,510,417.822 km^2^, distributed across five geographic regions (north, northeast, midwest, southeast, and south). In 2022, the country had approximately 203.1 million inhabitants, making it one of the most populous in the world [21].

### 2.4. Variables and Indicators

The selected variables include vaccine type, dose administered, year, state, and country. The vaccination schedule considered in this study was the same as recommended by the NIP for children up to one year of age, including the administration of the following vaccines: Bacillus Calmette–Guérin (BCG) and hepatitis B at birth; pentavalent (covering diphtheria, tetanus, pertussis, hepatitis B, and Haemophilus influenzae type B) and inactivated poliovirus vaccine (IPV) at 2, 4, and 6 months of life; 10-valent pneumococcal vaccine and human rotavirus vaccine at 2 and 4 months; yellow fever vaccine at 9 months; meningococcal C conjugate vaccine at 3 and 5 months; and the measles, mumps, and rubella (MMR) vaccine at 12 months of life [20].

In this study, coverage was considered complete when the final dose of each vaccination schedule was administered, except for the MMR vaccine, which was assessed only for its first-dose coverage, following the monitoring procedures adopted by the Brazilian Ministry of Health. For BCG and hepatitis B vaccines, which are indicated at birth, the single dose was considered [20].

### 2.5. Statistical Analysis

Vaccination coverage rates were estimated by considering the total number of last doses administered in a given year for each specific vaccine schedule (numerator) and the number of live births in the same period (denominator), multiplying the resulting ratio by 100 to present the outcome as a percentage. As a reference, the targets established by the National Child Vaccination Calendar under the NIP were used, namely: 90% for BCG and human rotavirus vaccines; 95% for all other vaccines [20].

To examine trends in childhood vaccination coverage in Brazil during the post-COVID-19 era, an interrupted time series (ITS) analysis was employed. This statistical technique is used to assess the impact of an intervention or event on a time series, particularly in scenarios where randomization is not feasible [18]. Since the event considered in this study—the COVID-19 pandemic—occurred at a well-defined point in time, it was possible to separate the period into pre- and post-event intervals.

To evaluate the effect of the COVID-19 pandemic on childhood vaccination coverage trends, the pre-pandemic period was used as a control, capturing what would have happened in the absence of the event. Thus, the assessment of the event’s impact aimed to examine any statistically significant changes that occurred in the post-pandemic period compared to the pre-pandemic period [18].

ITS involves modeling the time series as a function of time, incorporating underlying trends and seasonality when applicable. The “interruption” is then modeled as an abrupt or gradual change in the level or slope of the series, allowing for the estimation of the causal effect of the intervention.

We used Prais–Winsten regression with a single breakpoint in 2020, the year the COVID-19 pandemic began. Prais–Winsten is a modification of the Cochrane–Orcutt method designed to correct first-order autocorrelation in the residuals of a linear regression model. The general form of the equation was as follows:

*log*(*y_t_*) = *β*_0_ + *β*_1_*time_t_* + *β*_2_*D_t_* + *β*_3_*posttime_t_* + *ε_t_*, *ε_t_*∼*AR*(1)(*ρ*),

where *time_t_* is the calendar year coded 1 … 15 (2010–2024); *D_t_* is a dummy that equals 0 before 2020 and 1 from 2020 onward (immediate level change); and *posttime_t_* takes the values 0, 1, 2,… after 2020 to capture the change in slope. Parameters *β*_2_ and *β*_3_ therefore estimate, respectively, the abrupt shift in coverage level and the alteration in the annual trend attributable to the pandemic.

Prais–Winsten regression is useful for time series analyses in which autocorrelation of the residuals may violate the assumptions of ordinary least squares (OLS), resulting in biased estimates. By transforming the original equation, this method adjusts the data to address autocorrelation, leading to more precise coefficient estimates. The procedure is iterative and continues until residual autocorrelation is minimized. The Durbin–Watson test was applied to verify the correction of serial autocorrelation. This test yields values ranging from 0 to 4, with a value near 2 indicating an absence of autocorrelation, values near 0 indicating strong positive autocorrelation, and values near 4 indicating strong negative autocorrelation.

To address heteroskedasticity in the residuals and to obtain regression coefficients interpreted as annual percentage change (APC) with corresponding 95% confidence intervals, we performed a logarithmic transformation of the vaccination coverage values. Because the data were collected annually, it was unnecessary to include terms to model seasonality. We adopted a 5% significance level for this study.

The analysis was conducted using R software version 4.3.1, and the “segmented” package was employed to identify breakpoints in the time series.

## 3. Results

This study examined the temporal evolution of childhood vaccination coverage in Brazil from 2010 to 2024. The results were structured to highlight trends observed in the pre-pandemic years (2010–2019), the direct impact of the COVID-19 pandemic in 2020, and the subsequent trend from 2021 to 2024.

The coverage rates of nine vaccines recommended for children under one year of age between 2010 and 2024 show fluctuations, with a notable declining trend for several immunizations. Vaccination targets were satisfactorily met for most vaccines until 2015, while 2016 was the first year in which the targets were not reached for five (5) of the nine (9) immunobiologicals assessed in this study (Table 1).

The BCG vaccine maintained rates above 95% until 2018, failing to meet the 90% target in only four years (2019–2021 and 2023), coinciding with the period in which none of the vaccines achieved their targets. The yellow fever vaccine failed to meet the target throughout the entire study period (Table 1).

Four vaccines—pentavalent, poliomyelitis, meningococcal C conjugate, and yellow fever—have consistently failed to reach coverage targets since 2016. Despite the failure to meet targets for most vaccines, there was a clear year-over-year increase in coverage from 2022 to 2024 (Table 1).

**Figure 1:**
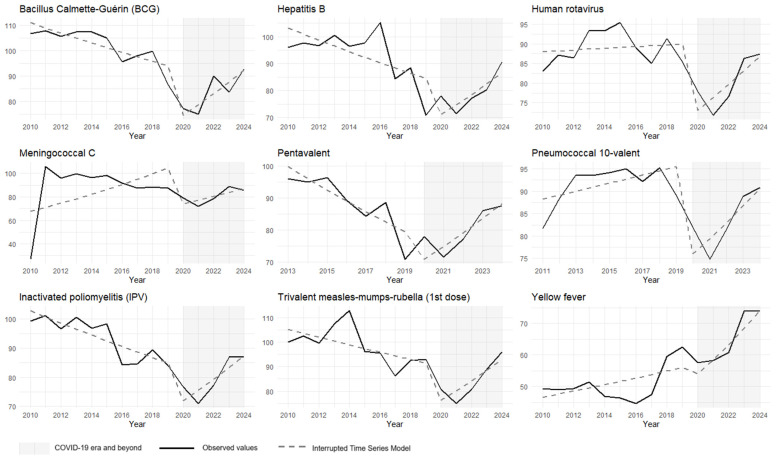
Interrupted time series analysis with Prais–Winsten regression model of vaccination coverage for the childhood immunization schedule (children under 1 year old). Brazil, 2010 to 2024. Source: National Immunization Program Information System. Note: coverage rates > 100% may reflect underestimation of the target population or vaccination of non-residents (under-registration of births, estimation errors, migration, etc.).

During the first period (2010–2019), vaccination coverage showed predominantly negative trends for five of the nine vaccines studied. Coverage for the pentavalent vaccine decreased at an average annual rate of −3.72% (*p* < 0.001), and hepatitis B coverage decreased by −2.21% per year (*p* = 0.015). Coverage for the inactivated polio vaccine and the meningococcal C vaccine also declined annually by −2.10% and −2.04% (*p* < 0.001), respectively. The remaining vaccines showed stagnation, with coverage oscillating around zero during this same interval (Figure 1).

The onset of the COVID-19 pandemic led to a sudden reduction in coverage for all vaccines analyzed, except for yellow fever. The most significant decline in 2020 was observed for the BCG vaccine, with a decrease of −24.88% (*p* < 0.001). The smallest reduction was for the pentavalent vaccine at −15.65% (*p* = 0.004), and yellow fever vaccine coverage decreased by −10.8%, which was not statistically significant (*p* = 0.296) (Figure 1).

From 2021 to 2024, the data suggest a gradual recovery in vaccination coverage. The coverage trends for BCG, hepatitis B, and polio vaccines became positive, increasing by 7.48% (95% CI: 5.23–9.79, *p* < 0.001), 7.45% (95% CI: 2.27–12.78, *p* = 0.014), and 7.24% (95% CI: 4.27–10.29, *p* < 0.001) per year, respectively. Similarly, the coverage for pentavalent, meningococcal, and measles–mumps–rubella vaccines showed average annual growth rates of 9.73% (95% CI: 7.30–12.23, *p* < 0.001), 6.46% (95% CI: 4.10–8.88, *p* < 0.001), and 6.73% (95% CI: 2.01–11.67, *p* = 0.017). The pneumococcal 10-valent and rotavirus vaccines also exhibited upward trends at more moderate rates of 3.47% (95% CI: 0.71–6.31, *p* < 0.001) and 4.12% (95% CI: 0.71–7.65, *p* = 0.036), respectively. The yellow fever vaccine did not change its pre-existing trend and continued to fluctuate around zero (Table 2).

Finally, the Durbin–Watson (DW) statistic, used to check for autocorrelation in the residuals of the fitted models, produced values close to 2, indicating that the Prais–Winsten model effectively corrected for serial autocorrelation.

## 4. Discussion

The findings of this study highlight a progressive decline in childhood vaccination coverage in Brazil between 2010 and 2019, with an exacerbation of this scenario in 2020 due to the impact of the COVID-19 pandemic. The sustained decline in essential immunobiologicals such as pentavalent, poliomyelitis, and meningococcal C vaccines points to structural challenges within the National Immunization Program (NIP). However, more recent data suggest a gradual recovery in coverage from 2021, particularly for BCG, hepatitis B, and MMR vaccines.

In the pre-pandemic period, consistent reductions in coverage rates for vaccines such as pentavalent, poliomyelitis, and meningococcal C already indicated low population adherence to routine immunization services. This scenario reinforces a misguided perception of the risk associated with vaccine-preventable diseases. This misconception is driven by the low incidence of confirmed cases and the absence of firsthand experiences with significant outbreaks of these illnesses. The success of the NIP from 1980 to 2010 in controlling and eliminating many of the diseases targeted by these vaccines has contributed to this false sense of security [22].

Also, the dissemination of false information discouraging vaccination was further amplified by the advent of infodemic, exacerbating vaccine hesitancy and creating additional barriers to maintaining high vaccination coverage, thereby exposing vulnerable populations to the risk of re-emerging previously controlled diseases [23].

Additionally, logistical and operational difficulties within the NIP may have worsening these negative trends. A recent survey conducted in state capitals in northeastern Brazil interviewed parents who, in contrast to the global trend of vaccine reluctance, overwhelmingly (99.1%) recognized the importance of vaccines for community health. In this context, it is essential to consider that non-vaccination is not exclusively associated with personal decisions but may also be influenced by external and structural factors, such as irregular vaccine supply, barriers to accessing services, limited operating hours, and economic hardships, among other challenges [24].

The impact of the COVID-19 pandemic intensified these dynamics. The sharp declines observed in 2020 for all analyzed vaccines, except yellow fever, can be attributed to various factors, including the temporary closure of vaccination services, mobility restrictions imposed by lockdowns, and the prioritization of COVID-19 response efforts at the expense of routine immunization activities. This phenomenon was not exclusive to Brazil but had global repercussions, as documented in other studies that demonstrate the interruption of essential services during this health crisis [25]. Even the BCG and hepatitis B vaccines, which theoretically should not have had their coverage affected during the pandemic as they are administered at birth, were affected. In Brazil, despite the recommendation of the Ministry of Health, vaccination in maternity wards is still not a widely practiced activity. Not all maternity wards in the country have adequate infrastructure to maintain vaccination rooms in continuous operation, especially those located in more remote regions or in small municipalities. The lack of an adequate cold chain, trained human resources, and basic equipment compromises the viability of the service. Vaccination in maternity wards requires efficient integration between hospital care and primary care, something that still has some weaknesses in the country. Many states and municipalities still do not have their own operational protocols or well-defined flows for vaccination to take place in maternity wards. In addition, those with lower vaccination coverage also tend to have greater logistical and operational difficulties in offering vaccines in maternity wards. Regional inequalities contribute to worsening the problem.

Furthermore, vaccine hesitancy, which had already been recognized as a growing issue [13], was intensified by the lack of assertiveness from the Brazilian federal government at the time in providing guidance to the population to deal with COVID-19 [26]. This favored the spread of fake news and drove political and social crises, as well as negatively impacting population adherence not only to COVID-19 vaccines but also to other immunizations within the national schedule [26]. The denialist discourse from the Head of the National Executive, the spread of misinformation, and the institutional weakening of the NIP fueled public distrust regarding the safety and efficacy of vaccines, increasing the risk of reintroducing previously controlled diseases [26,27].

A report from the U.S. Centers for Disease Control and Prevention (CDC) indicated that immediately following the emergency declaration of the COVID-19 pandemic in that country, there was a substantial reduction in the ordering and administration of routine pediatric vaccines [28]. A study conducted in Kenya used a methodology similar to that of the present study to quantify the pandemic’s impact on pentavalent and measles/rubella vaccine coverage and found that service disruptions affected routine immunization but noted a recovery in this indicator starting from the fourth month of the health crisis [29]. Another study, employing an interrupted time series (ITS) analysis, observed that the pandemic had a negative impact on the number of doses administered for the meningococcal C vaccine in some Brazilian states [30]. Our findings, alongside the cited literature, reinforce the need for differentiated, effective, and coordinated strategies not only to achieve vaccination coverage targets for children under one year old but also to immunize those who missed their vaccinations, thereby preventing the resurgence and outbreaks of previously controlled or eliminated diseases.

Despite the adversities faced during the pandemic, the gradual recovery of vaccination coverage from 2021 is a positive indication. In Brazil, several initiatives have been implemented to restore the previous culture of immunization and recover vaccination coverage. In 2023, there was a structural change in the Ministry of Health, including the reformulation and expansion of the NIP, which was upgraded from a General Coordination to a Department [31], granting it greater budgetary autonomy and visibility within the governmental structure. In the same year, the federal government launched the National Vaccination Movement, aiming to reverse vaccine hesitancy and expand access through educational campaigns and strengthened logistics. The mobilization included various immunizations, including the COVID-19 vaccine [32]. Additionally, nationwide microplanning workshops were conducted, involving SUS health teams from all Brazilian states and municipalities to enhance and qualify vaccination activities. Using a methodology developed by the Pan American Health Organization (PAHO), these workshops covered the following stages: health situation analysis, planning/programming, supervision with rapid vaccination monitoring, and evaluation [33].

As for the yellow fever vaccine, we observed that its coverage did not undergo significant changes over the study period. Historically, this vaccine was not universally recommended for the entire country, and was only recommended for areas at risk of transmission. This meant that parts of the Brazilian population were never included in regular campaigns or in the routine schedule, especially in coastal urban areas or in large urban centers in the south and southeast regions. With the re-emergence of cases and the spread of the virus to previously free areas, the Ministry of Health had to progressively expand the area where the vaccine was recommended. This gradual transition contributed to heterogeneity in vaccination coverage, with states and municipalities at different stages of implementation.

Finally, it is worth clarifying that in Brazil, monitoring of MMR vaccine coverage (measles–mumps–rubella) is conducted based on the number of first doses administered. Considering that the first dose is recommended at 12 months of age, the population used for the calculation (denominator) is the number of live births in the year. Therefore, we used the same methodology in this study. Vaccination coverage greater than 100% may seem contradictory at first glance, but it actually occurs for technical and operational reasons related to calculating coverage and the dynamics of health information systems. Considering that vaccination coverage is calculated by dividing the number of doses administered by the population and multiplying it by 100, if the estimate of the target population is below the actual number, the coverage rate may exceed 100%. This is common in municipalities with underreporting of live births, flaws in population estimates, and migration of families from other regions. Often, people who do not officially reside in the municipality get vaccinated there for convenience (work, travel, easier access). This increases the numerator, but these people are not included in the denominator, since they are counted as residents of another location. The results of this study should be considered in light of their strengths and limitations. Notably, the exclusive use of secondary data, while widely employed due to their official nature, may contain registration inconsistencies that impact the accuracy of the presented estimates. Another relevant aspect is the inability to establish direct causal relationships between the analyzed factors and the observed results due to the study design. The use of aggregated indicators may obscure significant socioeconomic or demographic disparities within the analyzed populations. Factors such as income, access to healthcare services, and regional inequalities may influence vaccination coverage differently and, consequently, the epidemiological analyses conducted. Finally, the COVID-19 pandemic introduced unprecedented challenges to the global landscape, and the consequences of this period may have exerted variable influences on local contexts, which may not have been comprehensively captured by the utilized data. Thus, it is essential to consider that the generalization of results may be limited, requiring caution when extrapolating findings to other contexts or periods. These limitations underscore the need for careful interpretation and the development of new and more comprehensive studies.

Although this study’s findings suggest a gradual recovery in Brazilian vaccination coverage following the pandemic, the pace and extent of this recovery have varied across different vaccines. While the upward trends are encouraging, Brazil remains far from meeting the targets established by the NIP in line with the WHO recommendations. Despite promising progress, none of the vaccines analyzed have yet reached the NIP’s coverage goals, underscoring the need for sustained and expanded immunization efforts.

Long-term strategies are essential to address regional disparities, increase coverage among vulnerable populations, and enhance healthcare professional training to counteract vaccine misinformation. In the post-pandemic context, recommended measures include multivaccination campaigns, intensified communication efforts, and improved logistics for immunobiologicals. Combating misinformation through evidence-based educational initiatives and expanding access to vaccination services are also critical.

Significant challenges remain, requiring strengthened intersectoral collaboration, improved governance within the Unified Health System (SUS), and active community engagement in vaccination campaigns. Sustaining coordinated efforts among policymakers, healthcare workers, and civil society will be vital for upholding immunization policies, rebuilding public trust, and re-establishing Brazil as a global leader in public health. Achieving this goal demands strong political and technical commitment, grounded in the country’s current context and the lessons learned from the COVID-19 pandemic.

## 5. Conclusions

Between 2010 and 2019, childhood vaccination coverage in Brazil showed a progressive decline, which worsened in 2020 when the COVID-19 pandemic caused immediate reductions of up to 25 percentage points. Interrupted time series analysis confirms that most vaccines were already on a downward trajectory before the health crisis. From 2021 onwards, a significant recovery was observed for seven of the nine vaccines evaluated; however, until 2024, none consistently reached the targets set by the NIP.

This scenario indicates recent progress but also evident structural weaknesses. To restore and sustain safe coverage levels, it is essential to consolidate the reorganization of the NIP, ensure efficient supply and logistics, adopt micro-planning in territories, and intensify communication strategies to combat misinformation and vaccine hesitancy. Strengthening these fronts is indispensable for preventing the resurgence of vaccine-preventable diseases and ensuring the resilience of the immunization system in the face of future public health emergencies.

## Figures and Tables

**Table 1:** Vaccination coverage rates (%) for vaccines recommended for children under one year of age. Brazil, 2010–2024.

VACCINES	2010	2011	2012	2013	2014	2015	2016	2017	2018	2019	2020	2021	2022	2023	2024
BCG	106.7	107.9	105.7	107.4	107.3	105.1	95.6	98.0	99.7	86.7	77.1	75.0	90.1	83.8	92.8
HEPATITIS B	96.1	97.7	96.7	100.6	96.4	97.7	105.2	84.4	88.5	70.8	77.9	71.5	77.2	80.3	90.7
HUMAN ROTAVIRUS	83.0	87.1	86.4	93.5	93.4	95.4	89.0	85.1	91.3	85.4	77.9	71.8	76.6	86.3	87.4
MENINGOCOCCAL C	-	105.7	96.2	99.7	96.4	98.2	91.7	87.4	88.5	87.4	79.2	72.2	78.6	88.8	85.8
PENTAVALENT	-	-	-	95.9	95.0	96.3	89.3	84.2	88.5	70.8	77.9	71.5	77.2	85.9	87.5
PNEUMOCOCCAL 10-v	-	81.7	88.4	93.6	93.5	94.2	95.0	92.2	95.3	89.1	82.0	74.8	81.5	88.9	90.8
POLIOMYELITIS (IPV)	99.4	101.3	96.6	100.7	96.8	98.3	84.4	84.7	89.5	84.2	76.8	71.0	77.2	86.9	87.1
MMR	99.9	102.4	99.5	107.5	112.8	96.1	95.4	86.2	92.6	93.1	80.9	74.9	80.7	88.9	95.9
YELLOW FEVER	49.3	49.0	49.3	51.5	46.9	46.3	44.6	47.4	59.5	62.4	57.6	58.2	60.7	73.9	73.9

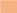
 Values below the vaccination coverage target; Source: National Immunization Program Information System. Note: Coverage rates >100% may reflect underestimation of the target population or vaccination of non-residents (under-registration of births, estimation errors, migration, etc.).

**Table 2:** Interrupted time series analysis using Prais–Winsten regression of childhood immunization schedule coverage: pandemic impacts and post-COVID-19 trends. Brazil, 2010–2024.

Vaccine Coverage	Trend 2010–2019	*p*	Change in Level ^b^ (COVID-19 Impact)	*p*	Trend 2020–2024	*p*	DW ^c^
	APC (CI 95%)		APC (CI 95%)		APC (CI 95%)		
Bacillus Calmette–Guérin (BCG)	**−1.83 (−2.47; −1.18)**	**<0.001**	**−24.88 (−30.55; −18.75)**	**<0.001**	**7.48 (5.23; 9.79)**	**<0.001**	1.916
Hepatitis B	**−2.21 (−3.70; −0.70)**	**0.015**	**−19.95 (−33.04; −4.30)**	**0.032**	**7.45 (2.27; 12.78)**	**0.014**	1.801
Pentavalent	**−3.72 (−4.82; −2.60)**	**<0.001**	**−15.65 (−22.30; −8.43)**	**0.004**	**9.73 (7.30; 12.23)**	**<0.001**	2.759
Inactivated poliomyelitis (IPV)	**−2.10 (−2.97; −1.22)**	**<0.001**	**−19.28 (−27.21; −10.49)**	**0.002**	**7.24 (4.27; 10.29)**	**<0.001**	2.059
Pneumococcal 10-valent	0.99 (−0.01; 1.99)	0.081	**−23.82 (−31.07; −15.82)**	**<0.001**	**3.47 (0.71; 6.31)**	**<0.001**	1.790
Human rotavirus	0.24 (−0.83; 1.34)	0.661	**−22.14 (−31.00; −12.14)**	**0.001**	**4.12 (0.71; 7.65)**	**0.036**	1.881
Meningococcal C	**−2.04 (−2.84; −1.24)**	**<0.001**	**−18.27 (−24.78; −11;20)**	**<0.001**	**6.46 (4.10; 8.88)**	**<0.001**	2.309
Trivalent measles–mumps–rubella ^a^	−1.53 (−3.03; −0.01)	0.074	**−20.74 (−32.19; −7.37)**	**0.014**	**6.73 (2.01; 11.67)**	**0.017**	1.839
Yellow fever	2.06 (−0.61; 4.82)	0.160	−10.80 (−27.29; 9.42)	0.296	5.97 (−1.38; 13.87)	0.142	1.553

APC = annual percent change. ^a^ refers to the first dose; ^b^ percentage variation in the time series level, measured immediately after the interruption, in 2020, which can be interpreted as an immediate impact of the COVID-19 pandemic on national coverage; ^c^ Durbin–Watson statistic. Bold text highlights values whose confidence interval does not include 0, and *p* < 0.05.

## Data Availability

The data and R codes from the analyses that supported the conclusions of this study are available upon reasonable request to the corresponding author.
